# Exploring the antibacterial, antidiabetic, and anticancer potential of *Mentha arvensis* extract through in-silico and in-vitro analysis

**DOI:** 10.1186/s12906-023-04072-y

**Published:** 2023-07-26

**Authors:** Shah Faisal, Muhammad Hamza Tariq, Riaz Ullah, Sania Zafar, Muhammad Rizwan, Nadia bibi, Aishma Khattak, Noora Amir, Abdullah

**Affiliations:** 1 Institute of Biotechnology and Microbiology, Bacha Khan University, Charsadda, 24460 Pakistan; 2 Department of Biotechnology, Virtual University of Pakistan, Lahore, Pakistan; 3 Department of Pharmacognosy, College of Pharmacy, King Saud University, Riyadh, Saudi Arabia; 4 Institute of Molecular Biology and Biotechnology, Bahauddin Zakariya University, Multan, Pakistan; 5 Center for Biotechnology and Microbiology, University of Swat, Swat, Pakistan; 6 Department of Microbiology, Shaheed Benazir Bhutto Women University, Peshawar, Pakistan; 7 Department of Bioinformatics, Shaheed Benazir Bhutto Women University, Peshawar, Pakistan; 8 Department of Physical Chemistry and Technology of Polymers, Silesian University of Technology, M. Strzody 9, 44-100 Gliwice, Poland; 9 Joint Doctoral School, Silesian University of Technology, Akademicka 2A, 44-100 Gliwice, Poland

**Keywords:** *Mentha arvensis*, Phytochemical analyses, Antibacterial, Anticancer, Antidiabetic, Molecular docking

## Abstract

**Background:**

*Mentha arvensis* has been utilized in diverse traditional medicines as an antidiabetic, anticarcinogenic, antiallergic, antifungal, and antibacterial agent. In this work, we have explored the phytochemical analyses and pharmacological potential of *Mentha arvensis* using both in silico and in vitro approaches for drug discovery.

**Methods:**

To determine the extract with the highest potential for powerful bioactivity, ethanol was used as the solvent. The phytochemical components of the extracts were quantified using liquid chromatography-mass spectrometry analysis. The potential bioactivities of extracts and lead phytocompounds, including their antibacterial, cytotoxic, and anti-diabetic effects, were evaluated.

**Results:**

The compounds oleanolic acid, rosmarinic acid, luteolin, isoorientin, and ursolic acid have been identified through liquid chromatography mass spectrometry analysis. Based on antimicrobial research, it has been found that the *Mentha arvensis* extract shows potential activity against *K. pneumoniae* which was 13.39 ± 0.16. *Mentha arvensis* has demonstrated a greater degree of efficacy in inhibiting α-glucosidase, with an inhibition rate of 58.36 ± 0.12, and in inhibiting α-amylase, with an inhibition rate of 42.18 ± 0.83. The growth of HepG2 cells was observed to be significantly suppressed upon treatment with extracts obtained from *Mentha arvensis*. Finally, In-silico methods demonstrated that the Luteolin and Rosmarinic acid exhibit acceptable drug-like characteristics. Furthermore, Molecular docking studies further demonstrated that both compounds have strong potential to inhibit the active sites of therapeutically relevant enzymes involved in Diabetes, Bacterial infections, and Cancer.

**Conclusions:**

The results of this study suggest that the *Mentha arvensis* extract possesses potent pharmacological potentials, particularly in terms of antibacterial, anti-diabetic, and cytotoxic effects. Particularly, Luteolin and Rosmarinic acid were identified as the top contenders for potential bioactivity with acceptable drug-like properties.

## Background

Mentha arvensis, commonly known as the wild mint, has been utilized by humans for its flavoring properties, as a decorative element, and for its medicinal benefits since ancient times. It has been incorporated into various food preparations as a flavor enhancer, utilized as a garnish to add visual appeal to dishes, and used in the production of herbal teas. In addition, *Mentha arvensis* has been used to prepare infusions, decoctions, and distillates, which are liquid extracts obtained through different methods of extraction. These preparations are valued for their ability to extract and preserve the plant's beneficial compounds, such as essential oils and phytochemicals, for use in traditional medicines and natural remedies [1, 2]. *Mentha arvensis* is utilized for various purposes including its breath-freshening properties, ability to tonify the stomach, insecticidal effects, anti-infective and anti-inflammatory actions, as well as its potential as an antiemetic, diaphoretic, antispasmodic, analgesic, stimulant, and emmenagogue agent [3, 4]. *Lamiaceae* is a substantial family of medicinal plants comprising over 6000 distinct species worldwide [5]. The *Lamiaceae* family can be divided into two major subfamilies, *Nepetoideae* and *Lamioideae*. The genus *Mentha* is classified within the *Nepetoideae* subfamily [6]. One of the many plant families, the *Lamiaceae* is utilized as a framework to assess the presence of several common secondary metabolites [7]. There are many terpenoids and phenolic chemicals among the usual secondary metabolites of the *Lamiaceae* [8]. Between 25 and 30 species in the genus Mentha are found in temperate South Africa, Australia, and Eurasia [9]. Mentha is used as a vast source of production of essential oil on a worldwide scale. *Mentha spicata, Mentha piperita,* and *Mentha arvensis* are extensively farmed around the globe in addition to the rapidly expanding populations, mostly for their output of essential oils [10, 11]. Additionally abundant in phenolic compounds are Mentha species, particularly phenolic acids and flavonoids [12, 13]. Typically, the phenolics and essential oil constituents of mint species are where the majority of their biologic activities come from [14, 15]. Data from the World Health Organization (WHO) shows that the traditional herbal remedies are used by 80% of the population in underdeveloped nations to treat their fundamental medical conditions [16]. At least 25% of pharmacological medications come from plants [17]. Compounds having pharmacological action may be found in a number of medicinal and aromatic plants [18, 19]. Certain medicinal and aromatic herbs have been shown to exhibit anti-septic, anti-carcinogenic, antiviral, antiallergic, anti-inflammatory, analgesic, estrogenic, and immune system boosting properties in recent investigations [20, 21].

An essential medicinal plant from the *Lamiaceae* family is *Mentha arvensis L*. The herb has stimulating, carminative, and fragrant properties [22]. It is estimated that 11.8 M tons of menthol is produced globally each year. The crude oil produced by *M. arvensis* accounts for the majority of output (9400 M tons). The top producer and exporter of maize mint oil is India, which also produces and sells menthol crystals, dementholized oil, mint terpenes, and other related items [23]. *M. arvensis* sometime known as maize mint or menthol mint, is mostly grown in northern and northwest India [24]. In countries like Bangladesh, India, Nepal, Sri Lanka, Japan and Thailand it is extensively farmed for usage as a culinary seasoning, a home treatment, and for industrial applications. This plant is reported to contain a variety of various chemicals, including menthol, menthofuran, neomenthol, isomnethone, d-menthone, pinene, menthylacetate, isomenthol, carvomenthone, phellandrene, cineol, limonine, p-cymene, carvacrol, piperitone an aromadendrene [25–28]. In traditional terms of medicine, *M. arvensis* is used as an anti-spasmodic, anti-peptic and carminative ulcer agent as well as used for the treatment of skin conditions, indigestion, colds and coughs. The plant is said to offer a variety of therapeutic benefits in various regions. The leaves have a variety of medicinal properties, including those listed below: thermogenic, stimulant, acrid, antihelmenthic, antispasmodic, febrifuge, deodorant, contraceptive, sudorific, dentrific, vulnerary, anodyne, carminative, digestive, diuretic, expectorant, cardiotonic, and hepatalgic [29]. The extract of plant shown to have antioxidant, hepatoprotective, anti-inflammatory, sedative-hypotonic, anti-allergic and antibacterial effects in recent studies [29–31]. According to different reports, *M. arvensis* possesses different number of therapeutic properties, including anti-inflammatory, anti-allergic, anti-fungal, and anti-bacterial properties [32]. The bulk of the food and beverage industries employ synthetic *M. arvensis* extract or flavours in dietary goods [33]. Results against certain cell lines indicated that *Mentha* spp. May have anticancer properties as well. Additionally, certain common species including *M. longifolia, M. piperita* and *M. arvensis* contain hydro-distilled essential oils that have shown substantial inhibitory actions against breast cancer in human cell lines MCF‐7 [34]. The objective of this research is to extract bioactive compounds from the ethanol extract of *Mentha arvensis* and conduct a comprehensive compound analysis. Due to its versatility in dissolving a variety of chemicals, high extraction efficiency, stability, safety, and regulatory approval, ethanol is frequently employed to prepare extracts for biological activity research [35]. The study will also assess the potential anti-cancer, anti-diabetic, and antibacterial activity of the extract using in silico and in vitro methods.

## Materials and methods

### Plant collection and extracts preparation

In this study the aerial parts of *Mentha arvensis* were obtained from the local market of District Charsadda, Khyber Pakhtunkhwa, Pakistan, by taking all necessary permissions and licenses from the concerned authorities and local bodies. The plant was taxonomically identified as *Mentha arvensis* by Shah Faisal Once identified, the voucher specimen of the material was deposited in a publicly available herbarium, as per institutional guidelines. The deposition number for the voucher specimen is 1259. The collected ariel parts were properly cleaned with tap water, dried in the shade, and then cut into little pieces. These pieces were then soaked in 95% ethanol, followed by using a Soxhlet extraction device to heat and extract ethanol for three hours. The excessive solvent was removed under reduced pressure at 45 °C in a rotating evaporator. The crude extract was then maintained at 4 °C in a refrigerator for later use.

### Liquid chromatography–mass spectrometry (LC–ESI–MS)/HPLC analysis

A Nexera HPLC system (Shimadzu, Japan), equipped with the double pump (LC-30AD), Auto sampler and column oven, was used to conduct the HPLC analysis (SIL-30AC). The column was a Chromolith RP-18 column (60 mm length, 5.6 mm ID) from Merck. Solvents A (0.3% formic acid aqueous form) and B (acetonitrile) were utilized to produce slope for the mobile phase, and the following conditions were utilized for gradient elution: 0–5 min, 5—20% of solution B; 5—10 min, 25% of solution B; 10—15 min, 25—35% of solution B; 15—20 min, 45—100% of solution B; 20—25 min, 100% of solution B. The volume of the injection was 5 L, while the rate of flow used for the separation was set to be 0.5 ml/min.. The Agilent Triple Quad mass spectrometer, which is a component of the LC–MS apparatus (LC–MS QqQ-6410B Agilent Technologies), was coupled to the chromatographic system (1260 Infinity Agilent Technologies). Following are the different parameters set for MS: MSn spectra: + ve and -ve modes, MS range: 100–1200 Da, gas temperature: 325 °C, nebulizer gas pressure: 45 psi and capillary voltage: 4000 V.

### Antibacterial activity

#### Bacteria strains preparation

The Khyber teaching hospital provided four standard isolated Bacterial strains, namely; *Escherichia coli* (ATCC 9637)*, Klebsiella pneumoniae* (AIS 2007023)*, Staphylococcus* (BAA-1690) *and Klebsiella aerogenes strain* (NCDC 819–56)*.* All the strains were grown for one day (24 h) at 37 °C with agitation of 200 rpm in the Mueller Hinton broth (Merck, Germany).

#### Disc diffusion experiment for antimicrobial susceptibility

In the current study, Disc diffusion assay was used to conduct the initial prediction of antimicrobial susceptibility [36]. In the current study, Disc diffusion assay was used to conduct the initial prediction of antimicrobial susceptibility antimicrobial test. Bacteria were cultured on the nutrient agar plates with an inoculum size of 1 × 10^5^ CFU/mL. Blank sterile discs having 6 mm diameter were impregnated with 20μL of 200 µg/mL of extract and were placed on the solid surface of prepared agar plates. By measuring the width of the inhibition zone (IZ) surrounding the discs, antibacterial activity was assessed. The test was trice repeated. The mean zone of inhibition diameters (mm) formed by the ethanol extract were used to express the antibacterial activity. Same protocol was also followed for Ciprofloxacin and oxacillinoxyacillin, as the positive controls, as they are known standard antibiotics for Bacteria under consideration.

#### Determination of minimum inhibitory concentration (MIC) and minimum bactericidal concentration (MBC)

The microdilution method utilizing broth was employed to perform the Minimum Bactericidal Concentration (MBC) and Minimum Inhibitory Concentration (MIC) assays. Tween-80 (Sigma) was added to pure solution of physiological saline (0.8%) at a final volume of 0.5% (v/v) and *Mentha arvensis* extract was dissolved in it. In a 96-well microtiter plate, different dilutions were prepared, ranging from 10% to 0.125%. In each well, 100µL of a specific dilution was added, followed by the addition of equal volume of bacterial strain solution, in such a way that the final concentration of each strain was maintained to 10^5^–10^6^ CFU/mL. MIC was determined as the concentration of extract, at which microorganisms demonstrate no obvious growth, whereas the MBC was estimated as the concentration of extract required to kill bacteria [37]. Each experiment was carried out in triplicates.

### Antidiabetic activaity

The α-glucosidase and α-amylase inhibition assays were used to assess the antidiabetic efficacy of *Mentha arvensis* extract.

#### α-amylase inhibition assay

The Alpha-Amylase inhibition assay was performed according to the previously published method [38] with certain modifications. Stock solution of 1 mg/1 mL of *Mentha arvensis* extract was prepared in 0.02 M sodium phosphate buffer and different concentrations (25-400 µg/mL) were made by using this stock solution,. Acarbose was used as a positive control in same concentrations while DMSO was used as the negative control. The resultant solutions were then incubated at room temperature for 10 min. This step was followed by the addition of 250µL of 1% starch solution which was further incubated at 37 °C for 10 min. To stop the reaction, 500µL of DNA reagent was added. Samples were then placed on the boiling water bath for next 5 min, followed by cooling at room temperature. Optical Density (OD) was measured at 520 nm and the following formulae was used to calculate the percentage inhibition:

$$Percentage\,Inhibition=\left[\frac{{Abs}_{control}-{Abs}_{test}}{{Abs}_{control}}\right]\times 100$$

#### α-glucosidase inhibition assay

The α-glucosidase inhibitory activity of *Mentha arvensis* extract was evaluated by following previously published protocol [39] with few alterations. 50μL of Mentha arvensis extract at different concentrations (25-400 µg/mL), 125µL phosphate buffer (0.1 M, pH 6.8), and α-glucosidase were incubated together at 37 °C for 30 min. Same protocol was followed with Acarbose and DMSO as the positive and negative controls, respectively. After incubation, 20μL of 1 M 4-Nitrophenyl-β-D- glucopyranoside (substrate) was added to the reaction mixture, followed by another 30 min incubation at room temperature. Finally, 50μL of sodium carbonate (0.1N) was used to stop the reaction and OD was determined at 405 nm and enzyme activity was determined as percentage inhibition by using the following formulae:

$$Percentage\,Inhibition=\left[\frac{{Abs}_{Blank}-{Abs}_{test}}{{Abs}_{Blank}}\right]\times 100$$

### Anti-cancer prospective of plant extract against HepG2 human cell line

HepG2 human cell line ATCC (HB 8065) was cultured in RPMI 1640 medium supplemented with FBS (10%), 100 μg/mL streptomycin, and 100 unit/mL penicillin. At 37 °C, Cells were seeded in 96-well plate in an atmosphere that was humidified and contained 5% CO_2_. Cultured HepG2 cells were treated with 200 μg/mL of *Mentha arvensis* extract, and incubated for 48 h. Same protocol was followed for positive (Doxorubicin) and negative (DMSO) controls. MTT (4,5-dimethyl diphenyl tetrazolium bromide) biochemical in-vitro assay was conducted to determine the effect of extract on the viability of HepG2 [40]. MTT (4,5-dimethyl diphenyl tetrazolium bromide) biochemical in-vitro assay was conducted to determine the effect of extract on the viability of HepG2. For MTT assay, 8 × 10^3^ cells were seeded in each well of 96-well plate, followed by a 24-h incubation. Then, plant extract was applied to the wells and plate was again incubated for 24 h under the same conditions. At the end of incubation, 100µL of 0.5 mg/mL MTT solution was used to replace the culture media, followed by an immediate incubation at 37 °C for next four hours. Finally, DMSO was added after removing MTT solution to dissolve the developed crystals. The absorption was find out at 570 nm using ELISA reader to determine the percentage cell viability by using the following formulae:

$$cell\,viability\left(\%\right)=\frac{O\,Dof\,Control}{O\,Dof\,Sample}\times100$$

### In-silico analysis

#### Evaluation of drug-like properties

In the first step of in-silico experimentation, drug-like properties of all 05 characterized compounds were assessed using Swiss ADME server. Swiss ADME assesses the drug-like properties of a compound based on five different rules namely, Lipinski rule of five, Ghose rule, and Muegge rule. Each of these mentioned rules evaluate drug-like properties on different parameters [41], as shown in Table 1. Furthermore, Physiochemical properties (Number of rotatable bonds, Number of Hydrogen bond donors, and Number of Hydrogen bond acceptors) and Medicinal Chemistry related properties (PAINS Alert and Synthetic Accessibility) were also evaluated from same SwissADME software.

**Table 1:** Factors and their ranges, on which different rules of drug-likeliness evaluate a compound

**Rule**	**Parameters**	**Range**
Lipinski rule of five [42]	Molecular weight	< 500 Da
	Hydrogen bond Donors	< 5
	Hydrogen Bond Acceptor	< 10
	Lipophilicity of ClogP	< 5
Ghose filter [43]	ClogP	-0.4 to 5.6
	Molecular weight	160 to 480
	Molar refractivity	40- 130
	Total number of atoms	20- 70
Muegge rule [44]	Molecular weight	200 to 600,
	XLOGP	-2 to 5
	Total Polar Surface Area	≤ 150
	Number of rings	≤ 7
	Number carbon	> 4
	Number heteroatoms	> 1
	Num. rotatable bonds	≤ 15
	Hydrogen bond Acceptor	≤ 10
	Hydrogen bond Donors	≤ 5

#### Establishment of compounds library

Compounds having all parameters within the normal range of the examined rules of drug-likeliness, were further taken for molecular docking analysis. Initially, the 3D structures of selected compounds were retrieved in SDF file format by employing Pubchem database. The downloaded structures were open up in one single library by employing Molecular Operating Environment (MOE) software [45]. The same software was considered to prepare these compounds for the molecular docking analysis by performing steps including protonation via Protonate3D algorithm, and energy minimization by AMBER99 force-field. Same protocols have been followed in peviously published studies [46–48]. Other then test compouds, positive controls were also considered for all docking experiments. Acarbose and Doxorubicin were considered as positive controls, for anti-diabetic and anti-cancer related docking protocols, respectively. Whereas, Ciprofloxacin and Oxacillin were used as positive controls for the docking studies related to anti-bacterial properties. The mentioned compounds were taken as positive controls as same drugs were also tested in wet-lab experimentations.

#### Preparation of target proteins

For docking analysis, crystal structures of five different proteins were downloaded from the Protein Data Bank (PDB) database [49]. Among these proteins, Alpha-glucosidase (PDB ID: 4GQR) and alpha-amylase (PDB ID: 5NN5) were retrieved for anti-diabetic related docking protocols as both enzymes digest carbohydrates and enhances the level of postprandial glucose in body, therefore, they are known as suitable therapeutic targets of diabetes [50, 51]. Thymidylate kinase (PDB ID: 4GQQ) was considered for anti-bacterial docking experiments as this enzyme is involved in the biosynthesis of Bacterial DNA, making it an attractive target for anti-bacterial drug discovery [52]. Similarly, BRCA1 (PDB ID: 4Y2G) and PCNA (PDB ID: 1AXC) proteins were considered for anti-cancer related docking experiments as their up-regulation has been reported to be associated with prognosis and development of cancer [53, 54]. The protein structures were prepared for docking analyses through MOE software, which included the removal of any attached ligands and solvent molecules, protonation by Protonate3D algorithm and energy minimization via AMBER99 force-field. Active sites in the target proteins were also determined by MOE software, where an active site was defined from the coordinates of the ligand in the original target protein sites.

### Molecular docking

Once the ligands library and the protein of interests were ready to dock, the MOE software was used to conduct the molecular docking analysis. Parameters considered to perform molecular docking experiments were; Refinement: forcefield, Rescoring: London dg, Placement: Triangle matcher, and Retain: 10.

### Statistical analysis

Statistical Package for the Social Sciences (SPSS) Version 17.0 (SPSS, Cary, NC, USA) was adopted to perform the basic statistical analysis of the data, obtained from experiments, whereas, Graphs were prepared by employing simple excel latest version.

### Ethics approval and consent to participate

We confirm that all methods used in this study, including the collection and use of *Mentha arvensis* plants, were performed in accordance with the relevant institutional, national, and international guidelines and legislation. Specifically, all necessary ethical approvals were obtained from the appropriate regulatory bodies before the commencement of the study.

## Results

### Phytochemical components of the extract

**Fig. 1:**
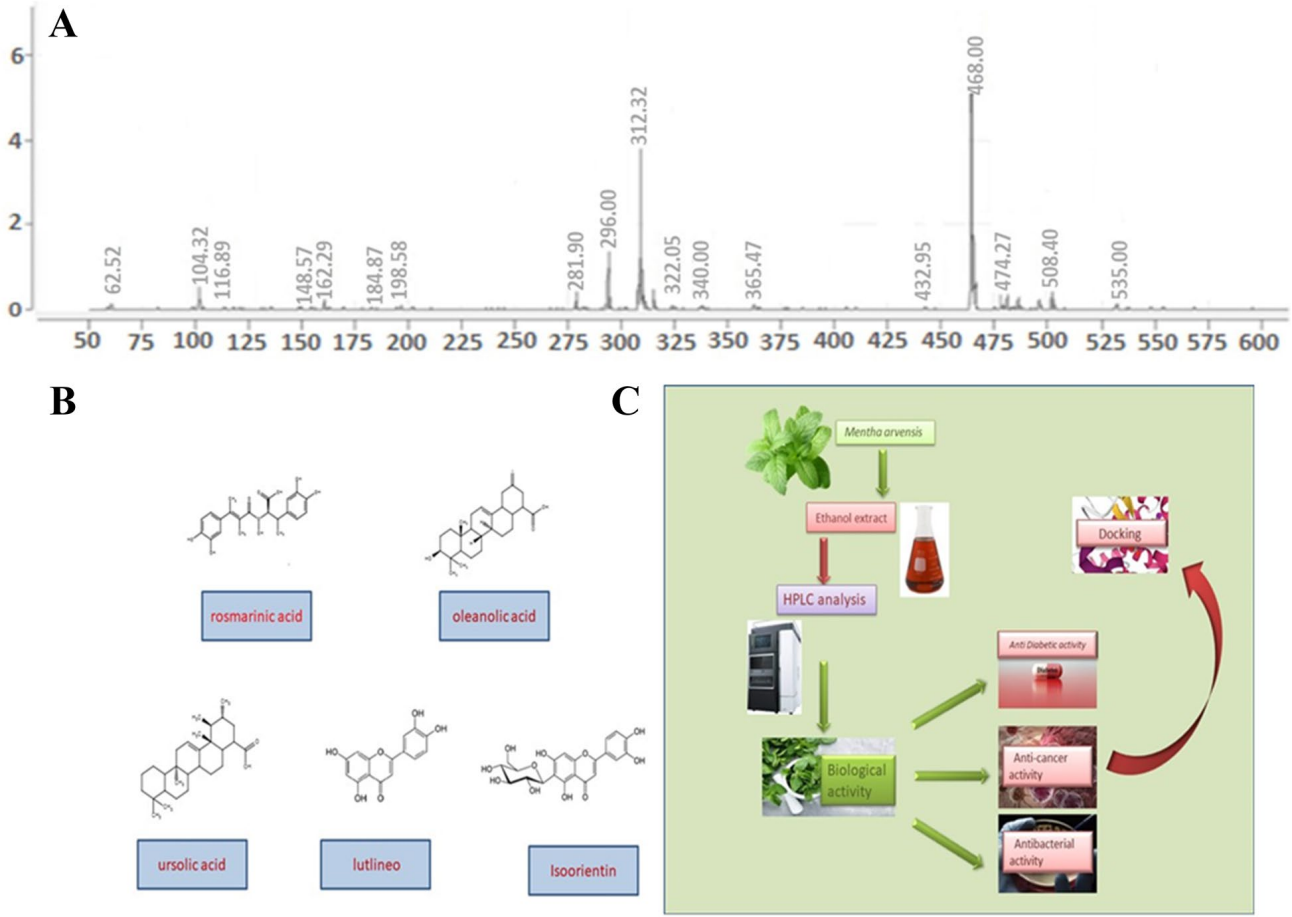
**A** LC–MS chromatogram of the ethanol extract of *M. arvensis* (**B**) Chemical compounds (**C**) Schematic illustrations of plant extract

The phytochemical analysis of the extract of *M. arvensis* was conducted using both LC–ESI–MS and HPLC–DAD techniques. The results, as presented in Fig. 1, which revealed the presence of several bioactive compounds (Fig. 1A) with Oleanolic acid, Rosmarinic acid, ursolic acid, luteolin, and isoorientin, being the richest ones, 2D structures of these compounds are demonstrated in Fig. 1B. Although for in-vitro biological assays, the whole extract was employed, yet, only the richest compounds were considered for the in-silico experiments. The schematic diagram of the entire methodology opted in the current research work is shown in Fig. 1C.

### Antibacterial assay

The ethanolic extract of *Mentha arvensis* was tested against four different pathegenic bacteria. Disc diffusion test helped in measuring the inhibition zone of *Mentha arvensis* extract discs and the results showed that the *Mentha arvensis* extract inhibits the growth of bacteria with inhibition zones of as 5.69 mm ± 0.49, 13.39 mm ± 016, 9.14 mm ± 0.42, and 7.42 mm ± 0.51 for *Escherichia coli, Klebsiella pneumoniae, Staphylococcus* and *Klebsiella*, respectively (Table 2). After the preliminary confirmation of antibacterial activity of extracts, a more detailed evaluation of *Mentha arvensis* extract for antibacterial activity was performed by measuring the MIC values. The MIC values were calculated by serial dilution, as the smallest amount of antimicrobials necessary to impede bacterial multiplication. The MIC values for *Escherichia coli, Klebsiella pneumoniae, Staphylococcus and Klebsiella* were found to be 3.2, 7.8, 4.5, and 5.1 µg/mL, respectively. This investigation also showed that the MBC values, which were found to be 6.2, 5.7, 4.6, and 2.8 µg/mL for *Escherichia coli, Klebsiella pneumoniae, Staphylococcus and Klebsiella aerogenes*, respectively.

**Table 2:** Antibacterial activity of *Mentha arvensis*

Bacterial Strains	Inhibition Zone (mm)	MIC (μg/mL)	MBC (μg/mL)
*Escherichia coli*	**5.69 ± 0.49**	**3.2**	**6.2**
*Klebsiella pneumoniae*	**13.39 ± 016**	**5.7**	**7.8**
*Staphylococcus*	**9.14 ± 0.42**	**4.5**	**4.6**
*Klebsiella*	**7.42 ± 0.51**	**2.8**	**5.1**

### Antidiabetic activity

Following anti-bacterial activity, the *Mentha arvensis* ethanolic extract was also tested for its ability to inhibit amylase and glucosidase enzymes, to estimate the anti-diabetic potential. For this purpose, different concentrations ranged from 25- 400 µg/ml were tested in in-vitro biochemical assays. Our research showed that the *Mentha arvensis* extract have concentration dependent alpha-glucosidase and alpha-amylase inhibitory action, as clearly indicated in Table 3.

**Table 3:** Antidiabetic activity of *Mentha arvensis*

**Conc. µg/mL**	**α-amylase**		**α–glucosidase**	
	***Mentha arvensis***	Acarbose	***Mentha arvensis***	Acarbose
400	42.18 ± 0.83	79.13 ± 0.46	58.36 ± 0.12	81.62 ± 0.21
200	28.25 ± 0.61	71.59 ± 0.48	51.32 ± 0.16	72.53 ± 0.53
100	19.37 ± 0.73	43.92 ± 0.52	31.44 ± 0.13	63.62 ± 0.37
50	10.36 ± 0.12	28.17 ± 0.20	24.18 ± 0.23	52.31 ± 0.79
25	7.29 ± 0.29	26.80 ± 0.28	18.21 ± 0.23	32.80 ± 0.21

### Anti-cancer activity

**Fig. 2:**
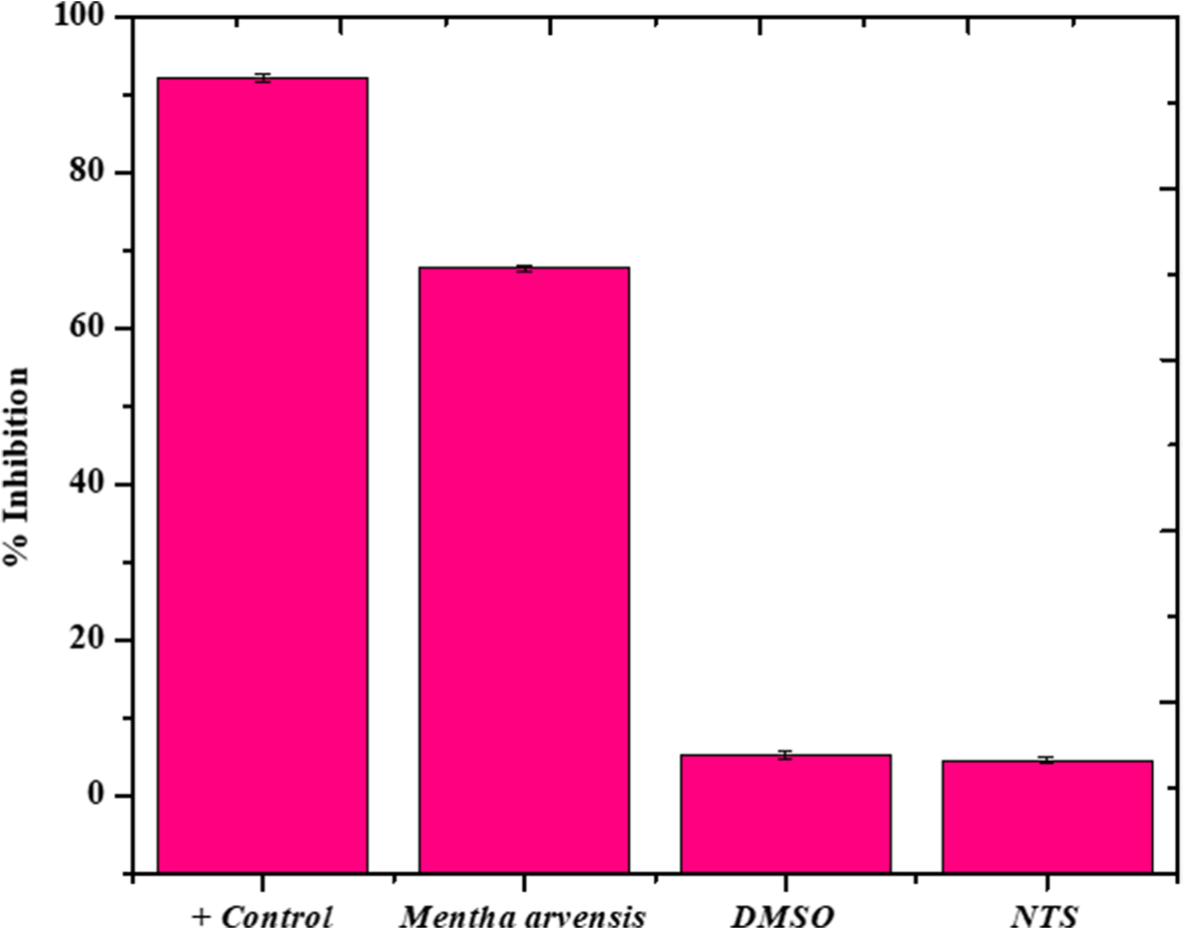
(**A**) Anti-cancer activity of *Mentha arvensis* extract

In the current research, the cytotoxicity level of *Mentha arvensis* extract was assesed in human liver cells (HepG2 cell line). When compared to the corresponding controls, HepG2 cells demonstrated a 67.73 ± 0.28 percent reduction following 24 h of *Mentha arvensis* extract treatment. Furthermore, the *Mentha arvensis* extract revealed significant death of cancer cells after a 24-h treatment period. The findings of this study demonstrated that *Mentha arvensis* extract had a very damaging effect on human hepatocytes, as seen in Fig. 2.

### In silico approach

#### Evaluation of drug-like properties

The purpose of this study was to propose some potential lead therapeutic compoundshaving acceptable drug-like properties, therefore, the characterized top five compounds from the Ethanolic extract of *Mentha arvensis,* were firstly scrutinized based on their drug-likeliness properties. SwissADME online server predicted that out of five only two showed all of the studied parameters within the normal range as shown in Table 3. These two phytochemicals are Luteolin and Rosmarinic acid and only these two compounds were taken further to retrieve the molecular docking results in comparison to the control drugs.

#### Molecular docking

**Fig. 3:**
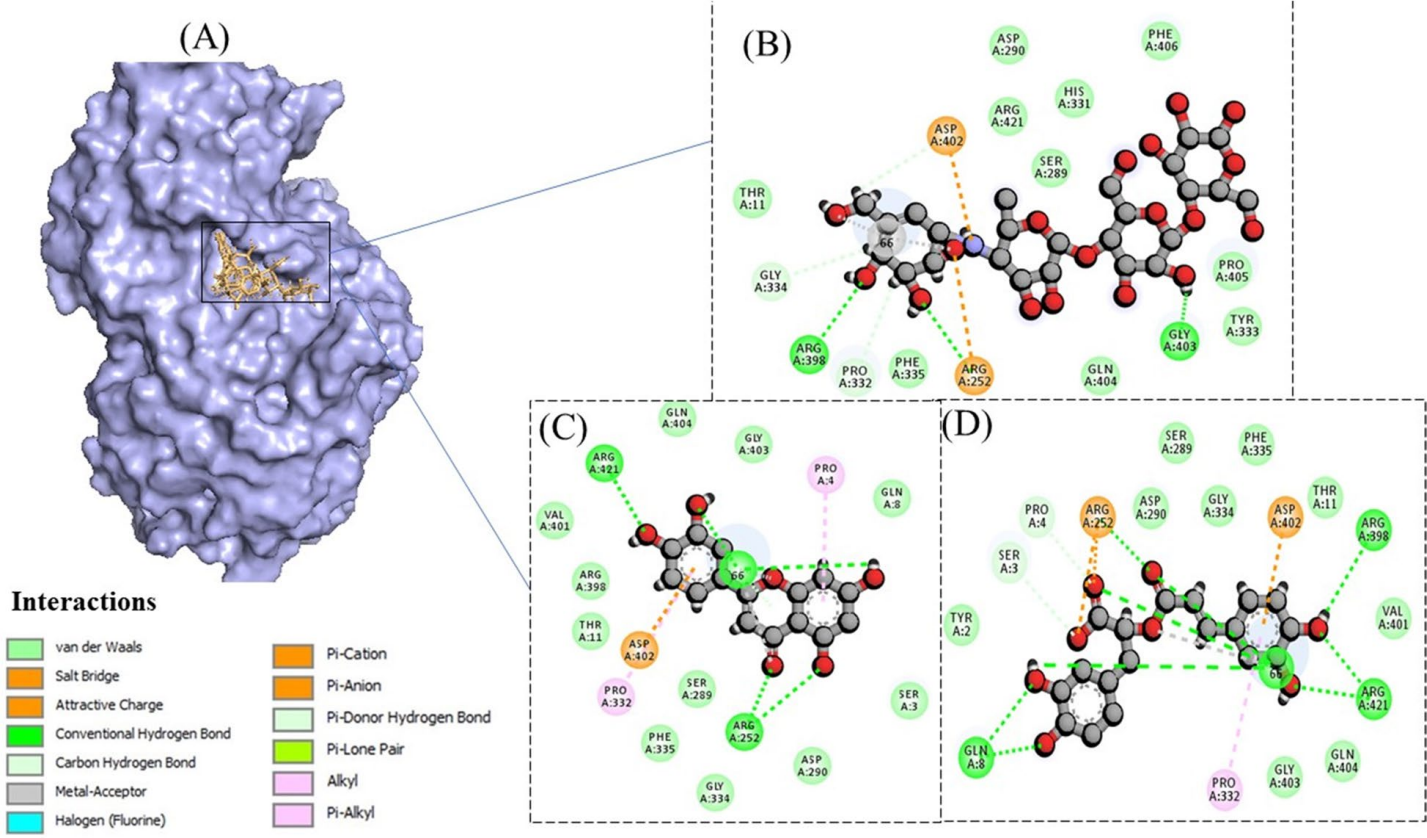
**A** 3-Dimensional representation of binding of compounds in the active site of alpha-amylase. Interaction processes are also demonstrated for Acarbose (**B**), Luteolin (**C**), and Rosmarinic acid (**D**). Different colors show different types of interactions, same color demonstrations are shown in other docking figures

**Fig. 4:**
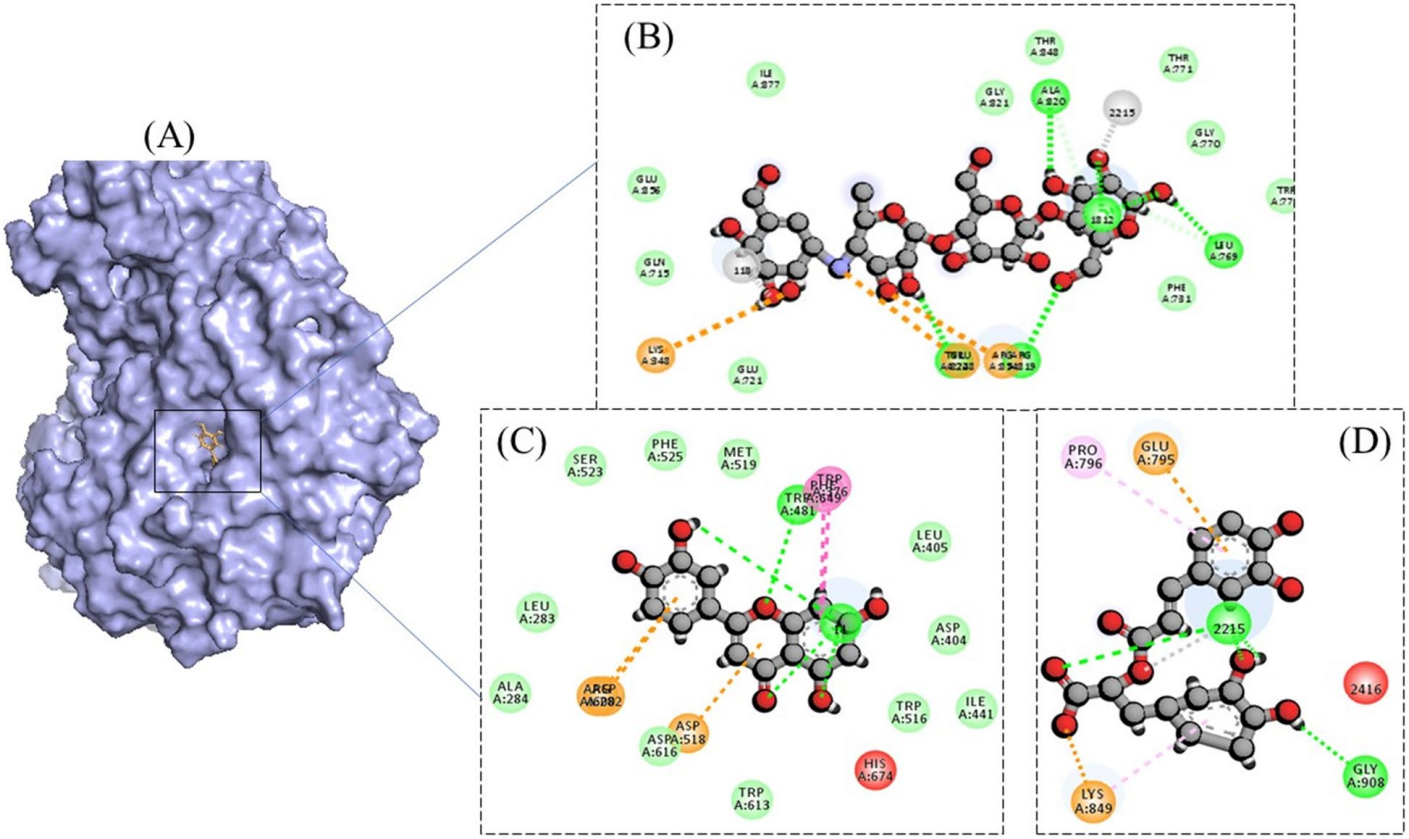
**A** 3-Dimensional representation of binding of compounds in the active site of alpha-glucosidase. Interaction processes are also demonstrated for Acarbose (**B**), Luteolin (**C**), and Rosmarinic acid (**D**)

In the wet-lab experiments, the ethanolic extract of *M. arvensis* was tested for anti-diabetic, anti-cancer, and anti-bacterial activities, therefore, enzymes reported to be the good therapeutic targets of these diseases were considered for in silico experimentation. Molecular docking was conducted along with the positive controls for each activity to compare the binding affinity of compounds of interests with the positive controls. MOE software showed that the binding affinity of the test compounds was either better or comparable to the positive controls in all of the studied cases, as demonstrated by docking scores and Root Mean Square Deviation (RMSD) values in Tables 4, 5, 6, 7. For alpha-amylase enzyme, the docking score of both Luteolin and Rosmarinic acid was lower than the acarbose (positive control), as shown in Table 4. Interaction studies showed that Acarbose (positive control) formed three hydrogen bonds with residues, Arg252, Arg398, and Gly403, while Luteolin formed four hydrogen bonds with residues Gly66, Arg421, Arg 252 (two bonds) (Fig. 3). Similarly, Rosmarinic acid formed nine hydrogen bonds, one with Arg 398, two with Gln 8, two with Arg 421, and three with Gly 66. For Alpha glucosidase, although the docking scores of test phyto-compounds was not better than the control, yet it was comparable (Table 4). Acarbose was found to be forming one hydrogen bond with Arg 219, Ala 220, Arg 238, Gly 312, and Leu 769 each. Contrary to this, both Luteolin and Rosmarinic acid were reported to be forming four hydrogen bonds. Where the former one formed bonds with Trp 481 and Gly 2215 (three bonds) and the latter one established bonds with Gly 908 and Gly 2215 (three bonds), as shown in Fig. 4.

**Table 4:** Physiochemical, medicinal chemistry and drug-likeliness associated properties of studied compounds from the ethanolic extract of *Mentha arvensis*

	**Physiochemical properties**					**Medicinal Chemistry**		**Drug Likeliness (Violations of Rules)**		
Compound name	Molecular Weight (g/mol)	Topological Polar Surface Area (Å^2^)	No. of rotatable bonds	Hydrogen bond donor	Hydrogen bond acceptor	PAINS Alert	Synthetic Accessibility	Lipinski Rule of 5	Muegge Rule	Ghose Rule
Oleanolic acid	456.70	57.53	1	2	3	0	6.08	1	1	3
Rosmarinic acid	360.31	144.52	7	5	8	1	3.38	0	0	0
Luteolin	286.24	111.13	1	4	6	1	3.02	0	0	0
isoorientin	448.38	201.28	3	8	11	1	5.04	2	3	1
Ursolic acid	456.70	57.53	1	2	3	0	6.21	1	1	3

**Table 5:** Binding energy and RMSD of luteolin, rosmarinic acid and acarbose with alpha amylase and alpha glucosidase enzymes, represented as docking scores and RMSD values

**Pubchem ID**	**IUPAC Name**	**Alpha Amylase**		**Alpha Glucosidase**	
		**Docking score (Kcal/mol)**	**RMSD refines**	**Docking score (Kcal/mol)**	**RMSD refines**
41,774	Acarbose (positive control)	-9.8915	2.3618	-12.5670	2.9579
5280445	Luteolin	-11.8655	1.7971	-11.5967	1.4267
5281792	Rosmarinic acid	-14.2675	2.7049	-11.8226	1.5566

**Table 6:** Binding energy and RMSD of luteolin, rosmarinic acid, ciprofloxacin, and oxacillin with thymidylate kinase, represented as docking scores and RMSD values

**Pubchem ID**	**IUPAC Name**	**Docking score (Kcal/mol)**	**RMSD refines**
2764	Ciprofloxacin (positive control)	-10.0786	0.5843
6196	Oxacillin (positive control)	-8.1645	1.2869
5280445	Luteolin	-10.6064	0.5439
5281792	Rosmarinic acid	-11.6084	3.3294

**Table 7:** Binding energy and RMSD of luteolin, rosmarinic acid and acarbose with BRCA and PCNA, represented as docking scores and RMSD values

**Pubchem ID**	**IUPAC Name**	**BRCA**		**PCNA**	
		**Docking score (Kcal/mol)**	**RMSD refines**	**Docking score (Kcal/mol)**	**RMSD refines**
31703	Doxorubicin (positive control)	-9.1847	3.6210	-12.6731	2.4280
5280445	Luteolin	-9.0922	1.4013	-9.2541	1.1543
5281792	Rosmarinic acid	-9.8695	2.3280	-11.7899	1.5829

**Fig. 5:**
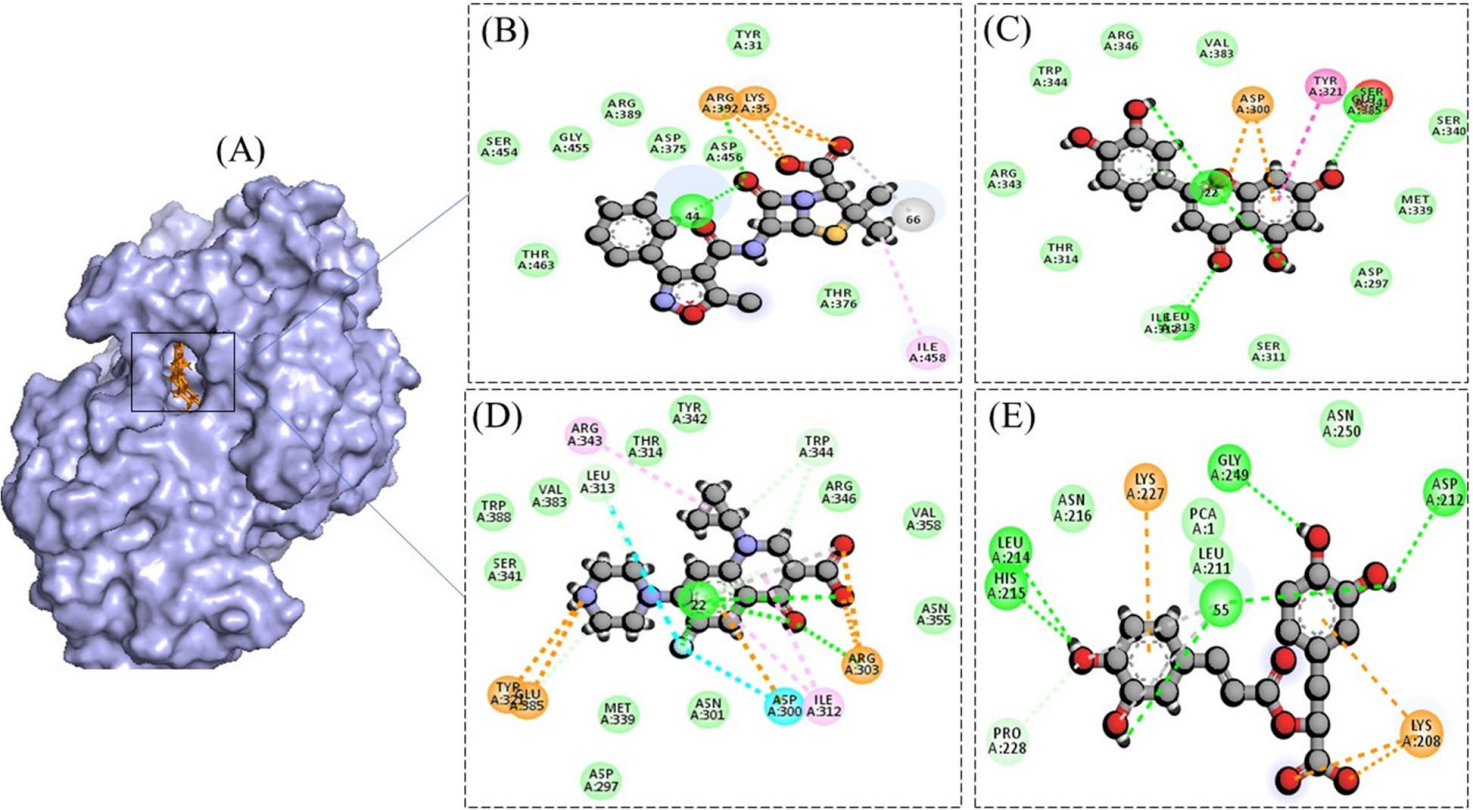
**A** 3-Dimensional representation of binding of compounds in the active site of Thymidylate kinase enzyme. Interaction processes are also demonstrated for Ciprofloxacin (**B**), Oxacillin (**C**), Luteolin (**D**), and Rosmarinic acid (**E**)

For Bacteria related docking experiments, Thymidylate kinase enzyme was taken as the target protein. Both of the under-consideration compounds demonstrated better docked complexes formation as compared to both of the studied control drugs (Table 5). Positive control drugs, Ciprofloxacin and Oxacillin, were reported to forming two and four hydrogen bonds, respectively. Ciprofloxacin constructed the bonds with Gly44 and Arg392 residues, whereas, Oxacillin developed single bonds with Leu213, Glu241 while two bonds with Gly22. Contrary to this, Luteolin established three hydrogen bonds with Gly22 and one with Arg303. On the other hand, Rosmarinic acid showed maximum number of Hydrogen bonds in this experiment as it formed one bond with Asp212, Leu214, His215, Gly249, and two with Gly55, as represented in Fig. 5.

**Fig. 6:**
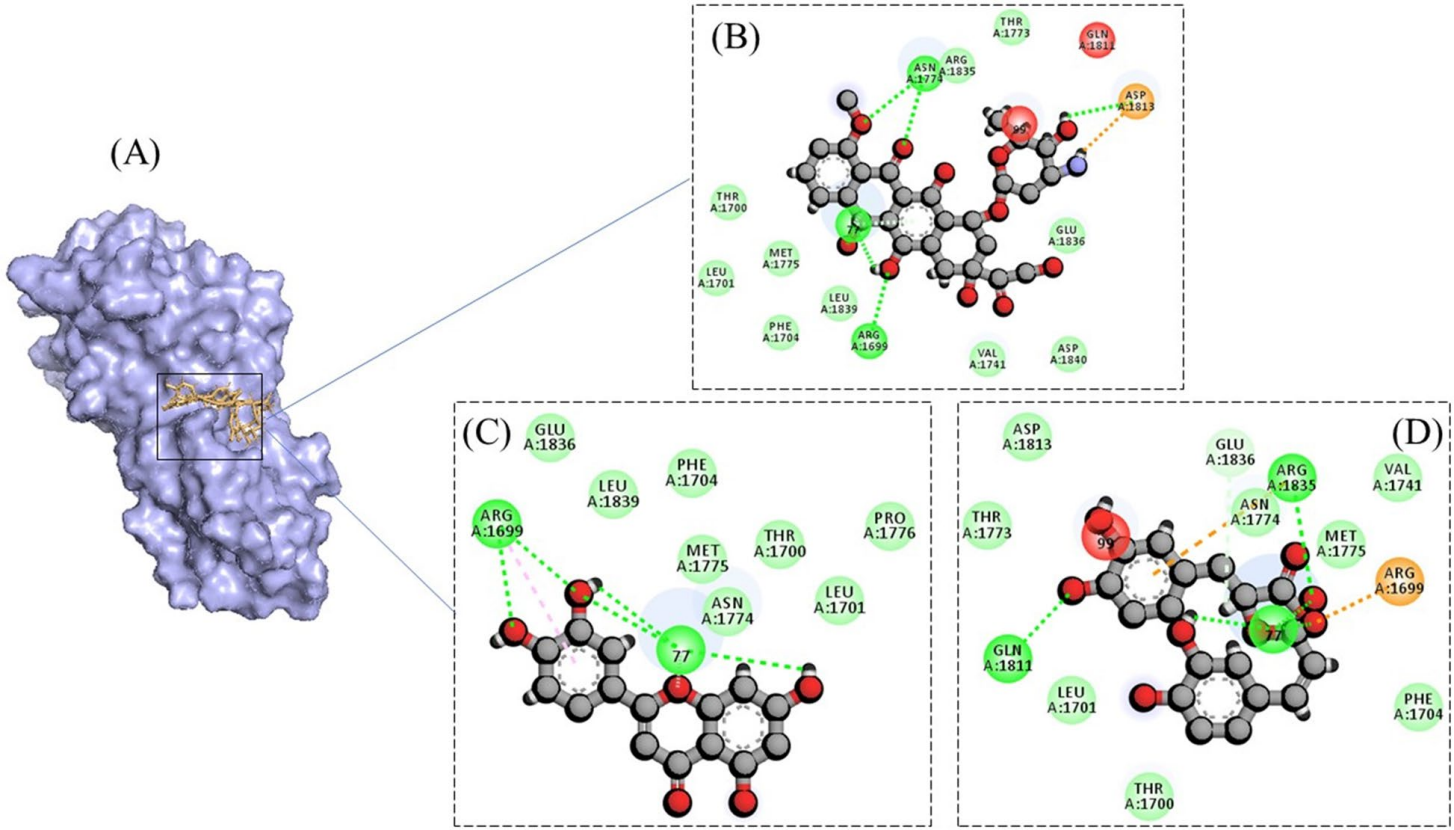
**A** 3-Dimensional representation of binding of compounds in the active site of BRCA protein. Interaction processes are also demonstrated for Doxorubicin (**B**), Luteolin (**C**), and Rosmarinic acid (**D**)

**Fig. 7:**
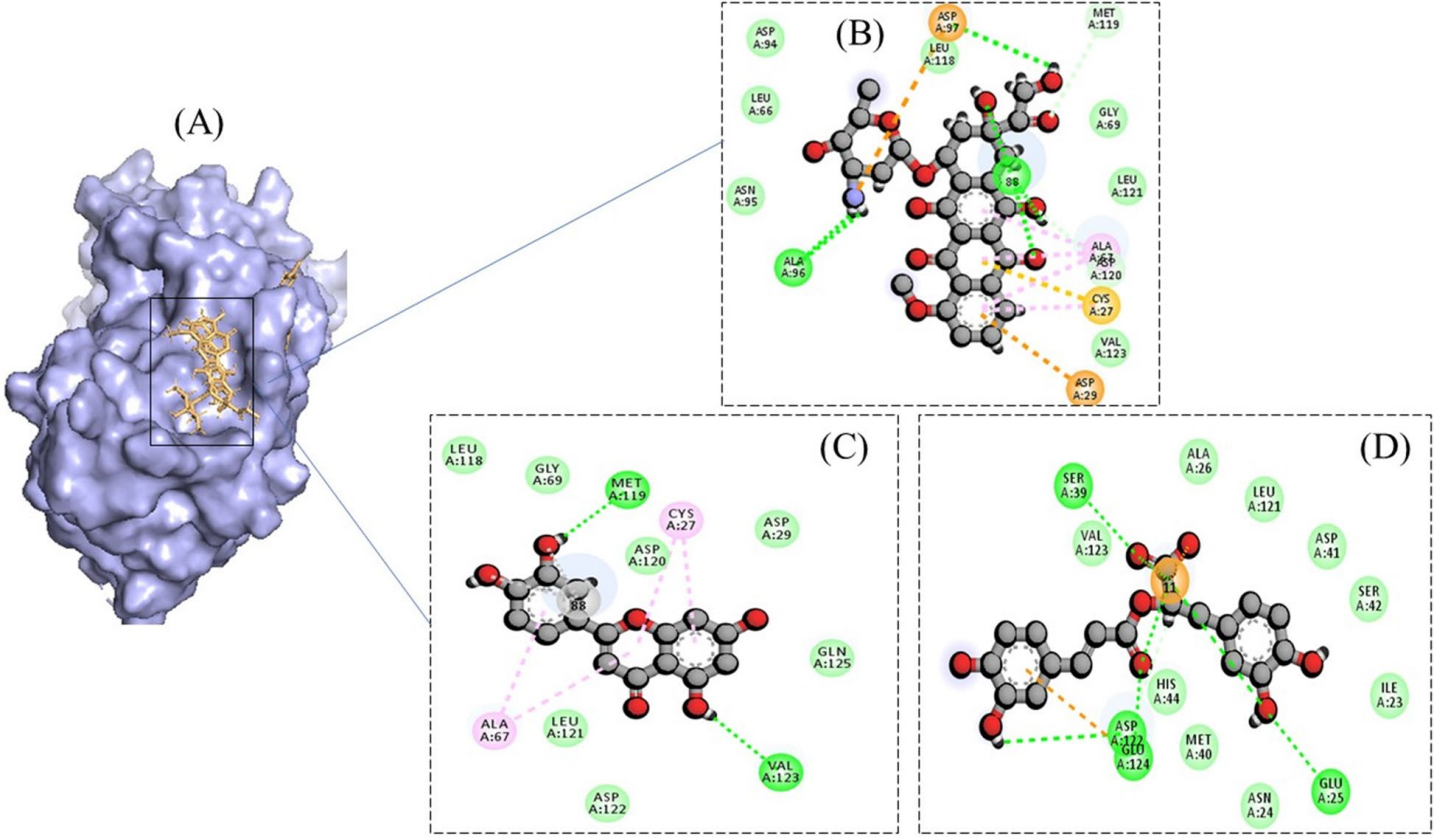
**A** 3-Dimensional representation of binding of compounds in the active site of PCNA protein. Interaction processes are also demonstrated for Doxorubicin (**B**), Luteolin (**C**), and Rosmarinic acid (**D**)

For anti-cancer experiments, the test compounds showed comparable docking scores with the positive control (Doxorubicin) for both target proteins, i.e., BRCA and PCNA (Tables 6 and 7). For BRCA, both Luteolin and Rosmarinic acid were seen to be having more Hydrogen bonds formation with the active site residues as compared to the control drug. Luteolin formed three hydrogen bonds with Gly77 and two with Arg1699, whereas Rosmarinic acid formed three Hydrogen bonds with Gly77, and one with Gln1811 and Arg1835 each. In comparison, Doxorubicin showed only four Hydrogen bonds, one with Gly77, Arg1699 and two with Asn1774 (Fig. 6). For PCNA, Luteolin demonstrated only two Hydrogen bonds (residues Met119 and Val123), Rosmarinic acid depicted six Hydrogen bonds (residues Glu25, Ser 39, Asp122, Glu124, and two with Asp11), and Doxorubicin represented seven Hydrogen bonds, out of which two with Asp97, two with Ala96, and three with Gly88 (Fig. 7).

## Discussion

The natural phenolic and flavonoid content of *Mentha arvensis* is enriched and plentiful, and numerous chemically active constituents may contribute to its anti-inflammatory and antioxidant effects [55]. This suggests that the crude medicines polyphenolic characteristic may have an antioxidant and anti-inflammatory impact. Investigating the phytochemical components and further assessing the anti-dibotic, antibacterial and anticancer capabilities of *M. arvensis* extracts were the goals of this work. The specific type of phytochemicals included in extracts that were identified by using high-performance liquid chromatography (HPLC). The HPLC findings showed The chemical components were isoorientin, luteolin, ursolic acid, rosmarinic acid, and oleanolic acid. Our findings are consistent with earlier publications [56].

Antibiotic resistance is a serious issue that has an impact on healthcare systems worldwide, both in developed and developing nations. Conventional antibiotic therapy have been significantly impacted by the development and spread of illnesses that are multidrug resistant [57]. Consequences indicate that the search for new antimicrobial drug suppliers has escalated in recent years in an attempt to tackle pathogenic illnesses that are treatment-resistant. Since they contain a variety of bioactive chemicals with well-known therapeutic characteristics, medicinal plants with antibacterial potential have been thoroughly investigated in this respect [58, 59]. The study showed that the ethanol extract of *M. arvensis* posses varying levels of antibacterial activity against every bacterium tested at various doses. All of the bacteria that were examined were extract-susceptible, with Klebsiella pneumoniae being the most susceptible with an inhibition zone of 3.39 ± 016. Our results were in line with those of other research that showed extract from *Mentha arvensis* has antibacterial activity [60].

To evaluate the anti-diabetic efficiency of *Mentha arvensis* extracts, cell-free, in vitro experiments of -amylase and -glucosidase inhibition at concentrations ranging from (25–400 g/mL) were carried out. Both -glucosidase (58.36 ± 0.12) and -amylase (42.18 ± 0.83) are more effectively inhibited by *Mentha arvensis.* Our findings concur with earlier publications [61].

We also investigated Aquilegia *Mentha arvensis'* ability to inhibit human hepatocytes cells (HepG2 cells). The tested extracts significantly decreased the viability of HepG2 cells, inhibiting their growth. Doxorubicin was useed as a + ve control, and it inhibited HepG2 cells by 92.51 ± 2.95%. On the other hand, aqueous plant extracts had a cytotoxicity or cell inhibition rate of 67.19 ± 9.2%. These findings suggested that extracts had an effective anti-cancer effect by lowering the viability of HepG2 cells. These extracts' cytotoxicity may be caused by three main pathways, involving the formation of ROS, the breakdown into functional components, and damage of DNA [62–64].

The second goal of this research work was to identify the putative therapeutic compounds within the extract of *Mentha arvensis* that could be considered as the future lead phytochemicals from *M. arvensis* for drug discovery. For this purpose, initially, drug-like properties of predominant phytochemicals were evaluated, from which it was found that the Luteolin and Rosmarinic acid were the only two compounds that show acceptable pharmacokinetics based on the studied parameters. Therefore, these two compounds were further taken for computational method of molecular docking experiments to check whether they can inhibit the therapeutical targets of different diseases, same method has been used in many previous studies [46, 65, 66] as molecular docking method explains the potential spatial and geometrical conformation in which a molecule or compound fits itself within the active site of target protein [67]. In this study, MOE software was used to perform molecular docking analysis, which predicts binding affinity in terms of docking score based on different parameters, such as van der Waal forces, rotatable bonds in ligands, hydrogen bonds, and desolvation electrostatic, same software is widely used to perform molecular docking research [68, 69]. Among the mentioned parameters, hydrogen bond plays the most critical role, more the number of these bonds is, the stronger the protein–ligand interaction will be as it results in the effective ligand displacement by causing rigorous geometric constraints [70]. MOE results represented that both Luteolin and Rosmarinic acid compounds demonstrated either better or comparable docking scores with the active sites of the studied proteins for all of the conducted experiments for different diseases, making both of them good therapeutically active phytochemicals for future studies, as proposed by other previously published studies which also propose the same based on the docking results [71, 72]. Both Luteolin and Rosmarinic acid have been previously reported to have numerous biological effects, including as anti-oxidative, anti-inflammatory, and anticancer activities, have been shown in earlier research [73, 74]. Therefore, our study not only further vaidates the therapeutic effect of these compounds, but also presents a novel natural source of their origin.

## Conclusions

The experimental findings in this study indicates that the ethanol extract of *Mentha arvensis* has promising potential as a candidate for various biomedical applications, owing to its notable antibacterial, anticancer, and anti-diabetic activities, as shown by the in-vitro experiments. Furthermore, in-silico experiments demonstrates that the *Mentha arvensis* extract under study contains two dominant phytochemicals, namely Luteolin and Rosmarinic acid, which possesses significant potential as lead therapeutic agents for the development of novel drugs targeting diabetes, cancer, and bacterial infections, however, further wet lab experiments are required to validate these findings.

## Data Availability

All data generated or analysed during this study are included in this published article.
