# Correction: Exploring the antibacterial, antidiabetic, and anticancer potential of *Mentha arvensis* extract through in‑silico and in‑vitro analysis

**DOI:** 10.1186/s12906-024-04398-1

**Published:** 2024-02-22

**Authors:** Shah Faisal, Muhammad Hamza Tariq, Riaz Ullah, Sania Zafar, Muhammad Rizwan, Nadia bibi, Aishma Khattak, Noora Amir, Abdullah

**Affiliations:** 1 Institute of Biotechnology and Microbiology, Bacha Khan University, Charsadda, 24460 Pakistan; 2 Department of Biotechnology, Virtual University of Pakistan, Lahore, Pakistan; 3 Department of Pharmacognosy, College of Pharmacy, King Saud University, Riyadh, Saudi Arabia; 4 Institute of Molecular Biology and Biotechnology, Bahauddin Zakariya University, Multan, Pakistan; 5 Center for Biotechnology and Microbiology, University of Swat, Swat, Pakistan; 6 Department of Microbiology, Shaheed Benazir Bhutto Women University, Peshawar, Pakistan; 7 Department of Bioinformatics, Shaheed Benazir Bhutto Women University, Peshawar, Pakistan; 8 Department of Physical Chemistry and Technology of Polymers, Silesian University of Technology, M. Strzody 9, Gliwice, 44‑100 Poland; 9 Joint Doctoral School, Silesian University of Technology, Akademicka 2A, Gliwice, 44‑100 Poland

**Correction:** ***BMC Complement Med Ther*** **23, 267 (2023)**

10.1186/s12906-023-04072-y

Following publication of the original article [1], the authors reported an error in affiliation for author ‘Muhammad Hamza Tariq’. His affiliation was changed from March 2023 and mistakenly shared the old affiliations (‘Department of Chemistry, National Taiwan University, Taipei, Taiwan’ and ‘Institute of Biological Chemistry, Academia Sinica, Taipei, Taiwan’). The correct affiliation for author ‘Muhammad Hamza Tariq’ is ‘Department of Biotechnology, Virtual University of Pakistan, Pakistan’.

The original article has been corrected.
